# Dynamic catcher for stabilization of high-viscosity extrusion jets

**DOI:** 10.1107/S1600576723003795

**Published:** 2023-05-29

**Authors:** R. Bruce Doak, Robert L. Shoeman, Alexander Gorel, Thomas R. M. Barends, Bogdan Marekha, Stefan Haacke, Stanislaw Nizinski, Ilme Schlichting

**Affiliations:** a Department of Biomolecular Mechanisms, Max Planck Institute for Medical Research, Jahnstrasse 29, 69120 Heidelberg, Germany; b Institut de Physique et Chimie des Matériaux de Strasbourg, University of Strasbourg – CNRS, Strasbourg, France; SLAC National Accelerator Laboratory, Menlo Park, USA

**Keywords:** serial crystallography, injection, high-viscosity extrusion, catcher

## Abstract

A ‘catcher’ based on a revolving cylindrical collector is described. The simple and inexpensive device reduces free-jet instabilities inherent to high-viscosity extrusion injection, facilitating delivery of microcrystals for serial diffraction X-ray crystallography.

## Abstract 2

A ‘catcher’ based on a revolving cylindrical collector is described.

## 1. Introduction

High-viscosity extrusion (HVE) injection was pioneered by Weierstall *et al.* (2014 ▸) to allow use of lipidic cubic phase (LCP) as a carrier medium (Liu *et al.*, 2014 ▸) for injecting membrane protein crystals into the X-ray beam of an X-ray free-electron laser (XFEL). XFELs deliver a pulsed X-ray beam of very short pulses (down to <1 fs) with a large number of photons (>10^13^) per pulse (Emma *et al.*, 2010 ▸). The short pulse duration temporally decouples the diffraction process from beam-induced structural changes (Neutze *et al.*, 2000 ▸). In serial diffraction methods, this ‘diffraction before destruction’ (Chapman *et al.*, 2014 ▸) therefore enables measurements on room-temperature macromolecular microcrystals with minimal radiation damage (Chapman, 2019 ▸; Barends *et al.*, 2022 ▸). The ensuing extension of XFEL techniques to proteins requiring a lipid environment, as offered by LCP, was a significant technical advance.

An HVE injector, like its low-viscosity predecessor, the gas dynamic virtual nozzle (GDVN) (DePonte *et al.*, 2008 ▸; Weierstall *et al.*, 2012 ▸), generates a free-jet fluid stream to transport the species of interest, embedded in a carrier medium, into the XFEL beam. Ideally, the jet should be no larger in diameter than the typical 1–5 µm diameter XFEL beam focus. However, passage of any heterogeneous medium through a conventional convergent nozzle with a diameter below about 50 µm leads to rapid and irrecoverable clogging. A jet of the required diameter therefore cannot be formed using a simple convergent nozzle. Consequently, GDVN and HVE injectors have no physical convergent nozzle profile; the free jet emerges from a fused silica capillary of uniform bore diameter along its entire length. In GDVN, gas dynamic forces exerted by a coaxially flowing gas function as a ‘virtual nozzle’, reducing the diameter of the low-viscosity free jet by a factor of up to 50 relative to the capillary diameter. This virtual nozzle does not clog. High-viscosity media, in contrast, cannot be shaped by gas dynamic forces. The virtual nozzle effect is absent and the extruding free jet essentially retains the diameter of the HVE nozzle capillary. Very high drive pressures are necessary to force a high-viscosity medium through small-diameter capillaries. The smaller the diameter and the longer the capillary length, the higher the required pressure. Aside from clogging, this places an additional stringent lower limit on the capillary diameter and thereby sets a lower limit on jet diameter and an upper limit on jet speed. To partially alleviate these constraints, Weierstall incorporated a pressure-amplifying piston into his injector and minimized the length of the nozzle capillary (Weierstall *et al.*, 2014 ▸). Current HVE capillaries typically have an inner diameter of 40–100 µm with a length of about 50 mm and produce jets ranging in speed from roughly 0.05 mm s^−1^ up to 10 mm s^−1^. These are essentially the fixed constraints of HVE injectors of the Weierstall design.

Nonetheless, it was soon recognized that the low speed of an HVE free jet can be quite advantageous. The jet speed depends on the viscosity of the medium, the diameter of the nozzle capillary and the volumetric flow rate of the sample medium through the HVE nozzle capillary. The speed is therefore easily adjusted by controlling the latter or, more specifically, by adjusting the flow rate of hydraulic fluid driving the pressure-amplifying solid piston inside the HVE injector (either flow rate or drive pressure can be chosen as the control parameter; generally flow rate is chosen, since it relates most directly to the volumetric flow rate of the jet and thereby to its linear speed).

For a carrier medium of given viscosity and with a judiciously chosen capillary diameter, the jet speed can generally be tuned within the range stated above. As it turns out, this range is extremely well matched to the 30 to 120 Hz pulse rates of first-generation XFELs. Since ‘diffraction before destruction’ of the XFEL pulse annihilates a segment of the sample jet, an undamaged jet segment must move into the interaction region before the next XFEL pulse arrives. The requisite jet speeds are easily calculated. If, for example, a 100 µm-long section of jet is damaged/destroyed, a jet speed of at least 1.5 mm s^−1^ is needed at 30 Hz pulse rate and at least 6 mm s^−1^ at 120 Hz. Both are well within the accessible HVE speed range. By properly tuning the jet speed, essentially all uncompromised sample in the jet can be probed, and this can arguably be defined as 100% sample usage. Not surprisingly, HVE sample injection has now expanded far beyond its LCP origins. A wide variety of high-viscosity carrier media have been vetted and found useful (Sugahara *et al.*, 2015 ▸, 2016 ▸ 2017 ▸, 2020 ▸; Botha *et al.*, 2015 ▸; Conrad *et al.*, 2015 ▸; Kovácsová *et al.*, 2017 ▸; Ihara *et al.*, 2020 ▸) and techniques for mixing biological crystals into these high-viscosity carriers have been developed (Sugahara *et al.*, 2015 ▸; Botha *et al.*, 2015 ▸; Fromme *et al.*, 2015 ▸). Accordingly, all such sample injectors are now generally termed ‘high-viscosity injectors’.

In diffraction before destruction, the jet is virtually stationary on the timescale of the XFEL pulse, so jet speed plays no role other than in setting the speed of sample replenishment. With more protracted X-ray pulses, this is no longer the case and jet speed becomes an active experimental parameter. Such is the situation in HVE measurements at synchrotrons (Botha *et al.*, 2015 ▸; Nogly *et al.*, 2015 ▸). A synchrotron produces a quasi-continuous X-ray beam but modern synchrotrons often allow a diffraction measurement to be gated, either by setting a finite frame rate on the detector or by use of a beam chopper. Yet all free-jet delivery is inherently gated by the transit of crystals through the footprint of the X-ray beam. The speed of the jet sets this transit time and offers a simple means of adjusting X-ray exposure even in the absence of explicit gating (and over a range of ×200, for the HVE speed range quoted above). This intrinsic gating must also be borne in mind if the measurement is to be gated by the detector frame rate or chopper, not just to correctly set the desired radiation dose but also to avoid experimental complications (multiple exposures of a single crystal, multiple crystals in a single exposure *etc*.). Since HVE injectors are easily incorporated into most synchrotron endstations, this ability to carefully control exposure per crystal and so limit radiation damage is a matter of some consequence, there being vastly more synchrotron endstations than XFEL endstations. Reduced damage, in turn, offers new possibilities for synchrotron measurements, such as diffraction measurements on room-temperature crystals including, at diffraction-limited storage rings (Eriksson *et al.*, 2014 ▸), time-resolved measurements on microsecond time scales.

### 1.1. HVE jet instabilities

As practitioners worldwide have discovered, HVE sample injection can be challenging. Stability of the HVE jet is perhaps the most demanding aspect of the technique. The jet tends to curl back on itself and ball up. The jet can slap back onto the nozzle to leave a viscous mass attached to the nozzle tip that disrupts extrusion. The free end of a vertical jet often oscillates side to side seemingly for no apparent reason, thereby moving the jet into and out of the X-ray beam. This behaviour becomes especially critical when multiple beams (*e.g.* X-ray plus pump and/or probe lasers) must be maintained in exact overlap with the sample free jet for an extended duration. These deleterious effects are more pronounced at higher jet speed and can be alleviated to some extent by surrounding the jet with a coaxial gas flow and by orienting the jet vertically downward, both of which are now standard HVE practices. The underlying cause is almost certainly electrokinetic charging of the sample medium as it passes through the HVE nozzle (Holstein *et al.*, 1999 ▸). The jet emerges electrically charged to one polarity and the nozzle to the opposite polarity, resulting in the free jet being attracted electrostatically back towards the nozzle. This induces slap-back onto the nozzle in the extreme case and transverse oscillations (restricted by the coaxial gas flow and/or by gravitational forces) in a milder manifestation. The faster the jet and the lower the mobility of the charged species in the jet medium (notably as limited by the high viscosity), the more pronounced the electrokinetic charging.

To limit these unwanted free-jet behaviours, a solid ‘collector’ or ‘catcher’ plate is generally positioned below the tip of the HVE nozzle. Viscous sample media adhere extremely well to solid surfaces. The HVE jet consequently attaches to and coils up on the collector surface, which both prevents slap-back onto the nozzle tip and also restricts side-to-side oscillations of the jet. Yet two new problems then arise: (1) The HVE material collected can pile higher and higher until it reaches the tip of the nozzle, physically blocking the jet flow in that manner. (2) Since newly arriving sample carries the same charge polarity as that of the material already deposited, the jet is electrostatically repelled from the accumulating pile. The contact point of the jet therefore wanders around the accruing pile, following a random path dictated by electrostatic forces. This wandering endpoint pulls the jet around with it, again displacing the jet relative to the X-ray beam. Sideways motion of the jet has been reduced but not eliminated. Applying suction through a hollow collector may assist matters, although very often the jet material then simply accumulates around the periphery of the suction opening. Yet even when the jet roams sufficiently little as to be usable for X-ray crystallography, its motion may preclude other types of measurements. We encountered this situation during HVE-based spectroscopy measurements in 2022. These measurements were extremely sensitive to stray light background, including light reflected from the jet itself and therefore influenced by motion of the jet. The method and device described herein were developed and found to markedly stabilize the jet, making such measurements possible but also being clearly of interest for HVE injection in general.

## 2. Results and discussion

### 2.1. Dynamic collection of the HVE jet

Our methodological innovation is to employ a moving rather than a stationary collector surface. The jet once again adheres to the collector but, rather than piling up, is carried away by the moving surface (see Movie S1 of the supporting information). A ‘scraper’ at some distance removed from the attachment point then shears the deposited material off the collector surface. The speed of the moving collector surface is set such that jet material is carried away at approximately the same rate at which it arrives. This then becomes a steady-state dynamical system, in which the jet flows steadily from the nozzle to the contact/attachment point on the moving collector surface, to be carried away as an attached ribbon on the rotor surface and then removed by the scraper. In this dynamic steady-state equilibrium, the attachment point of the jet on the collector is fixed in space, with the jet traversing a fixed path from the nozzle to this point. The jet is effectively pinned by these two endpoints. Moreover, there is no pile-up whatsoever of the charged viscous material on the collector at the attachment point. This eliminates both aforementioned problems (1) and (2), so the jet flows continuously and stably.

**Figure 1:**
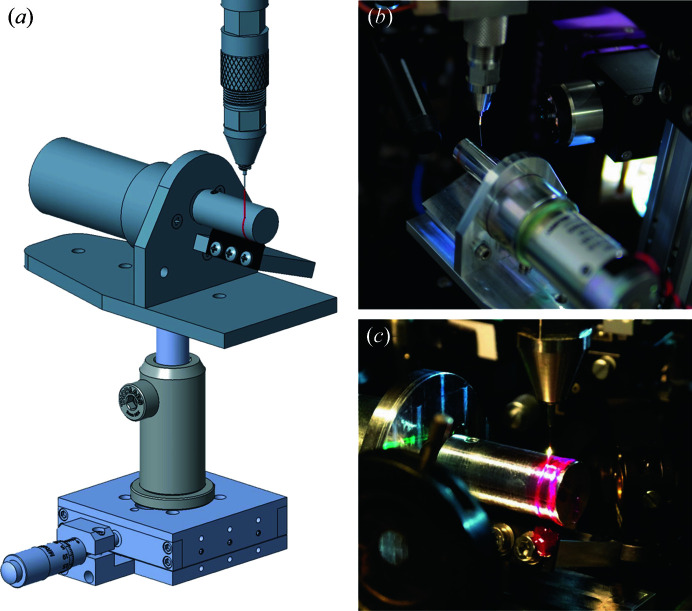
(*a*) CAD-construction drawing of the Rotating Rod HVE Catcher. Dynamically stabilized HVE collection in use for (*b*) crystallographic data collection at beamline ID29 at the ESRF and for (*c*) spectroscopy measurements on protein microcrystals embedded in viscous jets. The catcher was used to stabilize the injection of different protein microcrystals (ranging in size from 2 to 40 µm) embedded in LCP or SuperLube grease (Sugahara *et al.*, 2015 ▸). The compact design of the catcher is ideal for use in crowded environments such as goniometers and optical setups. Movie S1 of the injection at ID29 [shown in (*b*)] can be found in the supporting information.

To allow for a compact device, dynamic HVE jet collection is easily based on rotary motion, for example, by collection on (1) a rotating cylinder, (2) a rotating flat disc and (3) a looped conveyor belt. We have designed and fabricated versions of all three varieties, and used the first type in several variants at three different experimental facilities, for different purposes, under different environments and with different types of samples. The HVE jet was successfully stabilized in all cases. An image of this dynamic collector is provided in Fig. 1 ▸(*a*), taken from CAD-construction drawings of the device. The overall height of the assembly (excluding the HVE nozzle at the top) is 16 cm. A small, variable-speed DC gear motor (McMaster–Carr 2709K14, 19.1 VDC, 21 rev min^−1^ at 100 in-oz) turns a rod (or cylinder) of appropriate diameter, rotating it about a horizontal axis. The HVE sample stream flows vertically downwards from above to contact and adhere to the rotating rod near its top. By varying the DC motor drive voltage, the rotational speed of the motor is set to carry the arriving HVE sample stream away at approximately the same speed with which it arrives. The attached ribbon of jet material then rotates with the cylinder to arrive at a rubber blade, which scrapes the sample material off the cylinder. The device is simple but effective.

By switching the polarity of the DC drive voltage, the direction of rotation of the rotor can be reversed. Dual mounting holes allow the scraper to be shifted to the opposite side of the sagittal plane. The assembly can be shifted forwards and backwards relative to its support strut by use of different holes in the plate below the motor. Diverse experimental geometries can thus be easily accommodated. A translational stage (10 µm resolution) at the base of the assembly moves the assembly and rotor transversely with respect to the HVE jet, thereby setting the jet attachment point on the rotor. A simple sliding support post allows the cylinder to be moved up and down manually to set the vertical separation between the nozzle tip and rotor surface (millimetre precision suffices for this less critical positioning). To facilitate rapid construction of the seminal device, a simple design and readily available commercial parts were intentionally chosen. Optimization and sophisticated augmentations are clearly possible. However, this simple, robust device works so well that no such improvements have yet been deemed necessary.

Possible concerns are (1) detachment of the jet from the rotating rod, (2) momentary interruption of the nozzle flow or rupture of the jet between the nozzle and the collector surface, (3) variations in the jet speed or motor speed, and (4) stability of the scheme under diverse external forces acting on the jet. From our experience with this collector in experimental use, it appears that these are not a matter of great concern. Once attached, the jet does not detach.

An HVE jet is a viscoelastic medium and extensional strain along the jet axis can be relaxed through variations in jet diameter. Taking the jet radius *r* and jet speed *v* to be the critical variables, an incremental change d*l* of the jet length may be expressed as usual in terms of partial derivatives with respect to *v* and *r*, evaluated at the unperturbed values *l*_0_, *v*_0_ and *r*_0_, yielding d*l*/*l*_0_ = −2(d*r*/*r*_0_) + (d*v*/*v*_0_). Accordingly an incremental change in jet speed can be counteracted by an incremental change in jet radius that limits the change in jet length or even leaves it entirely unchanged. If the jet radius decreases, so too can the surface free energy of the jet, making this perturbation also energetically favourable. To accommodate both increases and decreases in jet speed, it is likely advantageous to operate under steady-state conditions that yield a slightly smaller jet radius than in the absence of the rotor, *i.e.* with the jet slightly ‘stretched’. Small decreases in jet speed can then be accommodated by a further increase in jet length, and speed increases by a relapse back towards the ‘unstretched’ length. Under these conditions, the jet is then not merely being ‘pushed’ out of the nozzle (via reservoir pressure applied by the piston) but also being ‘pulled’ (by the action of the rotor). This possibly contributes not just to spatial but also to speed stabilization of the jet. The latter, if verified, would be of great interest for time-resolved HVE measurements. This remains to be investigated experimentally, but certainly informed observation indicates that jet speed stability with the rotary catcher is at least as good as when using conventional catchers.

Velocity mismatch between jet and rotor surface can also be accommodated automatically by the ribbon attached to the rotor adopting a larger or smaller cross-sectional area than that of the incoming jet. Adaptation might also occur through the jet shifting its attachment point. All of these effects, plus viscous damping afforded by the HVE medium itself, are likely to contribute in making the rotor collection relatively insensitive to speed mismatch. In any event, optimal values for the rotor settings (rotational speed, vertical separation from nozzle to rotor surface, and horizontal offset of the nozzle relative to the rotor axis) can generally be found within a minute or two of manual adjustment.

Transverse jitter of the jet at the rotary catcher attachment point typically appears to be on the scale of a few millimetres. Jet jitter at the X-ray intersection point is generally at least a factor of ten smaller, since the X-ray beam intersects the jet a few hundred micrometres from the nozzle tip whereas the attachment point to the rotor is a centimetre or more below the tip. Moreover, by choosing the direction of rotor revolution to be along the X-ray beam rather than orthogonal to it, the jet jitters primarily back and forth within the beam rather than moving out of it.

A temporary interruption of the jet flow (*e.g.* due to passage of an air bubble through the nozzle or due to a momentary clogging event at the nozzle) does not appear to be critical. Provided the jet eventually does re-emerge from the nozzle and does grow in length to reach the rotating collector surface, it inevitably re-attaches. This is true even if the jet tip meanders about as it grows in length. Once the jet is re-attached, the system immediately returns to its steady-state dynamical equilibrium, with the jet stabilized.

### 2.2. Application of the rotating catcher

The rotating catcher was recently used in measurements at beamline ID29 (https://www.esrf.fr/id29#) of the ESRF synchrotron, Grenoble, France [see Fig. 1 ▸(*b*)], to stabilize injection of different protein microcrystals ranging in size from 2 to 40 µm. The crystals were embedded in LCP or SuperLube grease (Sugahara *et al.*, 2015 ▸). Fairly strong drafts were present in the hutch during our beam time, causing the jet to wave in the breeze before it attached to the rotating collector. However, once attached, it immediately snapped into a dynamic steady-state equilibrium and effects of the draft disappeared other than a slight quivering of the jet, perhaps driven by vortex shedding in the draft (Movie S1 of the supporting information). Hence it appears that the catcher can stabilize even an HVE jet subjected to strong, intermittent, physical forces.

Photographs of the jet and rotating catcher in operation are shown in Fig. 1 ▸, taken during its ID29 use [Fig. 1 ▸(*b*)] and during the spectroscopy measurements [Fig. 1 ▸(*c*)] mentioned earlier. Even on the crowded optical table of the latter, there was ample room for this compact catcher. The leftmost stripe on the rotating rod in Fig. 1 ▸(*c*) is the attached ribbon of the jet. The other, larger stripe closer to the end of the rod is due to overflow around the end of the scraper, which did not extend all the way to the end of the rotor in this instance. Still, the actual deposition path is scraped clean. The bright white spot on the downwards flowing vertical jet marks the point where a laser beam intersects the jet. There is ample clearance for this optical laser beam above the collector. In an XFEL measurement, the X-ray beam would intersect the jet at about the same point. An X-ray detector subtends a large solid angle relative to the interaction point, and it is critical that any HVE collector does not shadow the detector. By judicious choice of rotor diameter, jet attachment point and nozzle-to-rotor positioning, this constraint is easily met.

We have made no attempt to test the rotary catcher with the many HVE media that have now been proposed, not least because our testing and development were carried out during real experimental measurements that dictated the choice of media. Nonetheless our extensive experience with HVE injection (co-invention of the original LCP injector; continuing design, fabrication, and use of HVE injectors and catchers of all types in the subsequent years) suggests that most HVE-compatible media should function well with the rotary catcher. Some fine-tuning of the medium properties may be needed in specific cases, but this also is true of HVE injection with standard catchers. Optimum positioning of the catcher relative to the free jet must also be explored with every new sample and jet speed but, as mentioned above, this is easily and quickly done. Accordingly, we offer an enthusiastic imprimatur for this latest addition to our HVE family of devices.

## Supplementary Material

HVE injector and rotating catcher setup at beamline ID29 at the ESRF. DOI: 10.1107/S1600576723003795/te5116sup1.mov

Supporting figure and Movie S1 description. DOI: 10.1107/S1600576723003795/te5116sup2.pdf
