# Supplementary material for: Dynamic catcher for stabilization of high-viscosity extrusion jets
Source: J Appl Crystallogr. 2023 May 29;56(Pt 3):903–7. doi: 10.1107/S1600576723003795 (PMC10241051; doi:10.1107/S1600576723003795)
Supplement: Supplementary file 2 [file j-56-00903-sup2.pdf]

JOURNAL OF
APPLIED
CRYSTALLOGRAPHY

Volume 56 (2023)

Supporting information for article:

Dynamic Catcher for Stabilization of High Viscosity Extrusion Jets

R. Bruce Doak, Robert L. Shoeman, Alexander Gorel, Thomas R.M. Barends, Bogdan Marekha, Stefan Haacke, Stanislaw Nizinski and Ilme Schlichting

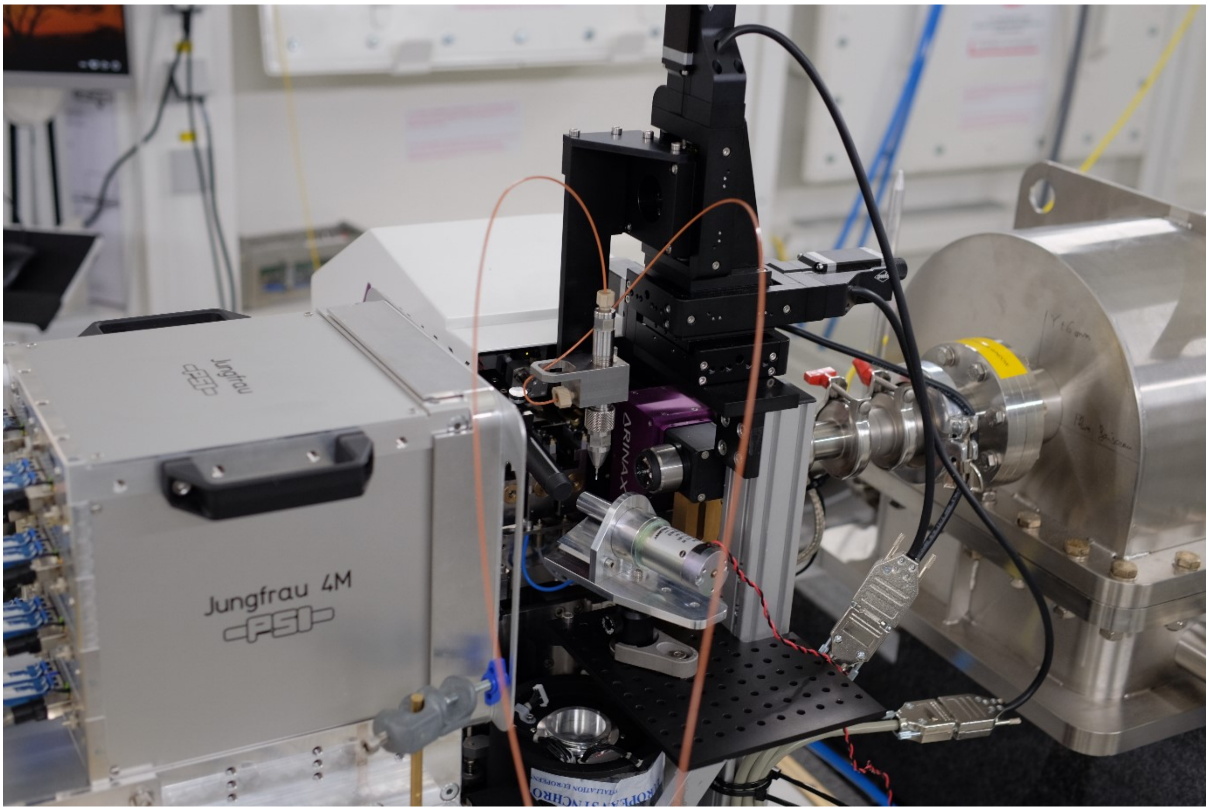

Supplementary Figure S1. Photo of the experimental setup at ID-29 of the ESRF, with which Supplementary Movie S1 was recorded. The Heidelberg "block-mount" HVE injector and dynamic catcher assembly are seen near the centre of the image. The X-ray beam passes from right to left through the scattering centre (just below the nozzle tip) and on into the detector. To accommodate other beamline components, the catcher in this case was not quite orthogonal to the X-ray beam.

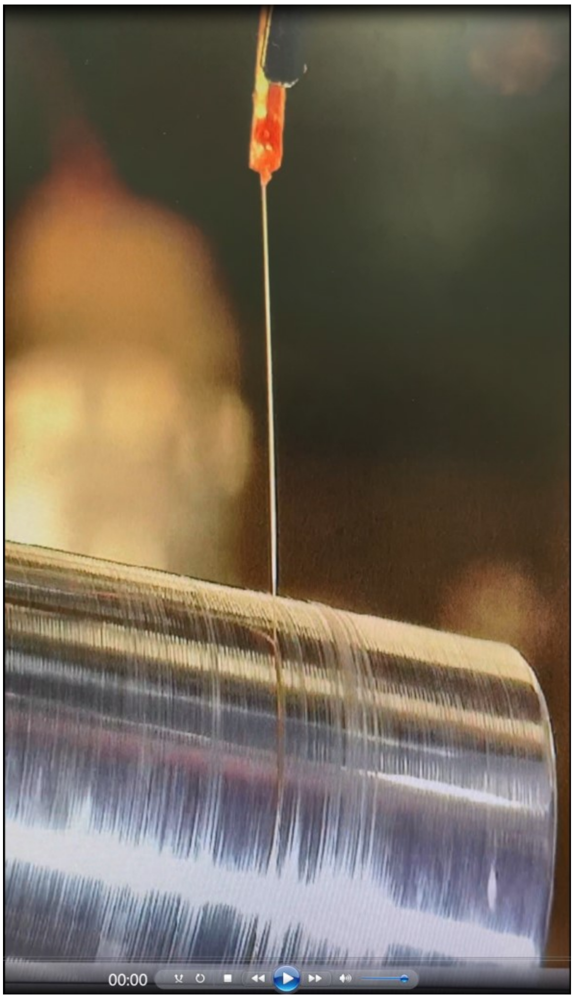

Supplementary Movie S1. HVE injector and rotating catcher setup at beamline ID29 of the ESRF. The rotor diameter is 14 mm. Microcrystals were embedded in LCP. The volumetric flow rate of fluid driving the piston inside the HVE nozzle was 10.0 µL. The pressure amplification ratio of this piston is 8.46 and so the average flow rate out of the nozzle capillary was 1.18 µL/min. Taking the diameter of the extruded jet to be equal to the 75 µm diameter of the nozzle capillary, the linear jet speed was 4.5 mm/s. The observed quivering of the jet is due to drafts in the hutch at the time of our experiment. Even this slight bowing introduces significant axial stress in the jet. Nonetheless, the jet did not rupture and remained firmly attached to both the nozzle and to a contact point on the rotor. The contact point shifts by no more than a millimetre or two, and this motion is clearly due to the drafts.
